# Patient Safety: Developing and Evaluating a Workshop for Preclinical Students

**DOI:** 10.1111/tct.70054

**Published:** 2025-02-26

**Authors:** Kirsty Matthews, Millie Pierce

**Affiliations:** ^1^ Warwick Medical School Coventry UK

**Keywords:** patient safety, medical students, peer and near‐peer education, professionalism

## Abstract

**Background:**

Patient safety is a core feature of undergraduate medical education, yet literature shows its implementation in curricula to be challenging and variable. Through the pilot and evaluation of an ‘Introduction to Patient Safety’ workshop, this project presents an initiative of how to address this curriculum challenge. Student‐patient collaboration was championed in workshop design and delivery, addressing a regrettable lack of patient involvement to‐date.

**Approach:**

Workshop activities were designed to hold authentic patient voice as central and to encourage interactivity. Storytelling was used to build empathy with the range of individuals involved in patient safety. Additionally, a gameshow‐inspired activity highlighted key ideas from our patient advisory group. Responding to evaluation, the session was adapted and delivered by student facilitators to a full cohort. Advice on how to act, should students observe a patient safety incident, was incorporated.

**Evaluation:**

Evaluation was comprehensive and multi‐faceted. Pilot workshop attendees participated in semi‐structured interviews, with the transcripts thematically analysed. Additionally, both authors produced a written reflection, and their academic supervisor fed back on the session recording. After full‐cohort delivery, questionnaire feedback was also collected; 88% of 117 respondents rated the session at least 4 out of 5 (5 = *very good*).

**Implications:**

The workshop has since been integrated into Warwick Medical School's core curriculum. Designed and delivered by medical students, this project has shown the significant impact medical students can have in contributing meaningfully to undergraduate curriculum development. Medical students hold a unique position within healthcare settings to be powerful drivers of patient safety.

## 1 Background

Patient safety is an integral component of undergraduate medical curricula, and a core feature of outcomes set by the General Medical Council for UK graduates [1]. The World Health Organisation have devised a patient safety curriculum for medical schools and embrace learning on this topic from Year 1 [2]. Nie et al note, however, that literature on patient safety initiatives in undergraduate medical education are limited, mostly targeting medical students in the later clinical phases of their education, rather than in earlier years [3, 4]. Early introduction, however, can improve learner attitudes and awareness regarding safety [4].

Through understanding this context and identifying a gap within the Warwick Medical School   curriculum, this project aimed to develop and evaluate an ‘Introduction to Patient Safety’ workshop for preclinical students. Now a regular feature in the curriculum, this workshop—designed by medical students collaborating with patients—highlights how students can positively enhance curriculum design and addresses a lack of existing patient involvement.

## 2 Approach

**FIGURE 1:**
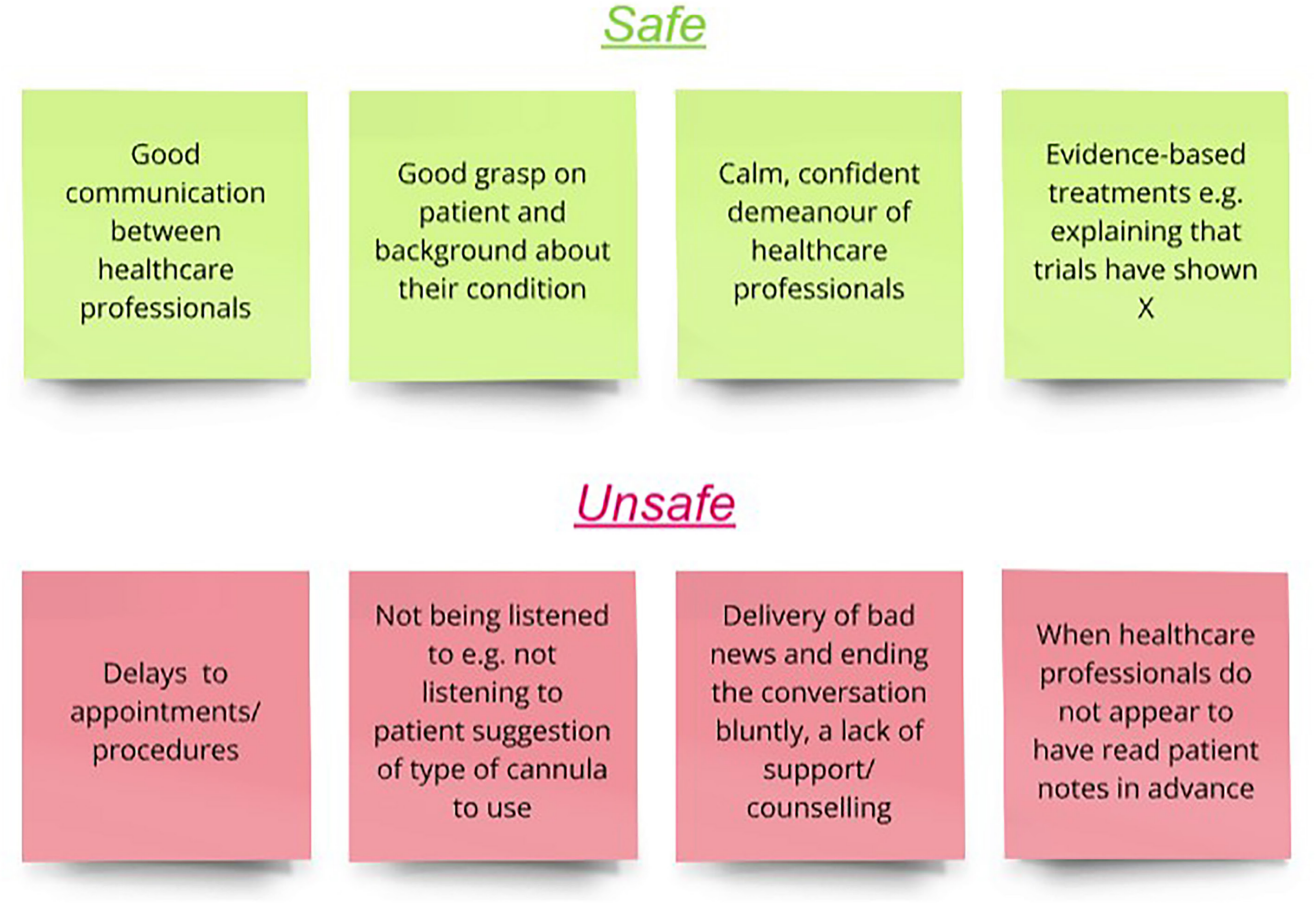
Sample responses from the Warwick Medical School patient advisory group when asked ‘What makes you feel safe or unsafe in a healthcare setting?’, displayed on a virtual whiteboard (Miro [6]).

Led by two graduate‐entry medical students, a two‐hour design session was held with the Warwick Medical School   patient advisory group, to inform workshop content. Questions surrounding what made patients feel safe and unsafe within healthcare environments and how healthcare professionals should act when an error has occurred were explored, using a virtual whiteboard, shown in Figure 1. Patient voice was valued and prioritised, respecting the active role that patients can play in determining patient safety [5].

A pilot workshop was held with five first‐year medical students in August 2022; several methods were used in its evaluation. Areas of improvement identified were used to make changes. The workshop was then delivered in November 2022, to all second‐year students, having completed their pre‐clinical stage and imminently commencing full‐time clinical placement. A total of 117 questionnaire responses were received after the session, including a Likert scale rating.

Student facilitators were recruited from the cohort, with previous experience in peer‐teaching desirable. A face‐to‐face training session was provided, along with a detailed facilitator guide, which highlighted the session learning objectives:

- Explain the importance of patient safety
- Recognise factors which contribute to patient safety in a healthcare environment
- Explain how healthcare professionals should act when something has gone wrong

The final workshop was delivered by 12 facilitators to groups of seven to eight and was structured as follows:

- **Introduction:** highlighting the importance of patient safety, via a mini quiz
- **Gameshow‐inspired activity:** students were asked to identify the most important factors in helping the patient group feel safe
- **‘In your shoes’ storytelling exercise:** this exercise was inspired by ‘A Mile in My Shoes collection,’ developed by the Health Foundation and the Empathy Museum [7]. A video of a patient speaking about a patient safety incident was shown [8]. Students were encouraged to tell the stories of different individuals involved in her care, leading to a group discussion
- **Warwick Medical School   student stories:** discussion of anonymised accounts of incidents that students encountered on placement and an overview of management
- **Practical advice and signposting of available support:** tips for students, if they experienced a concerning situation, and details of available support

## 3 Evaluation

**FIGURE 2:**
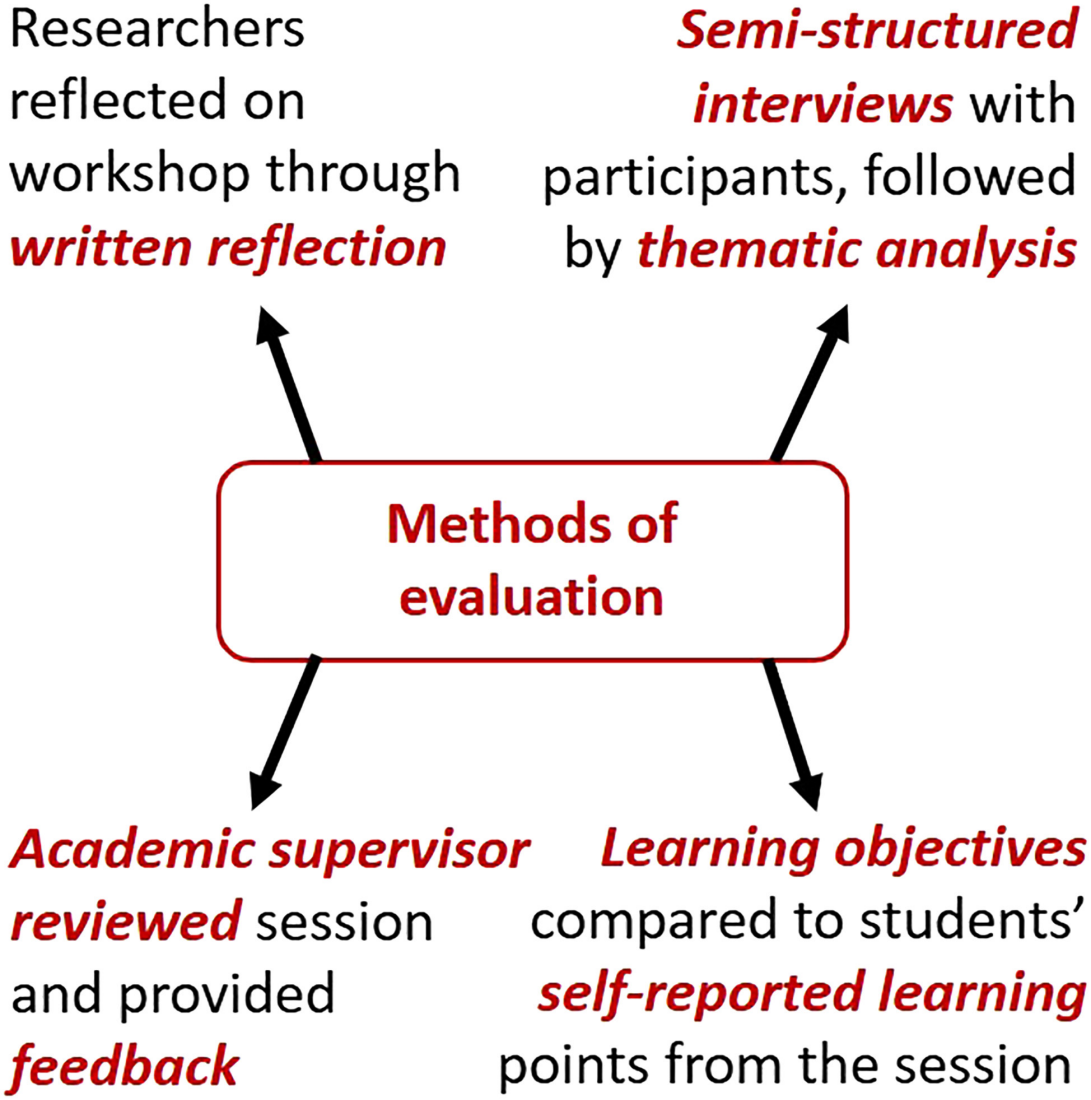
Methods of evaluation used.

Evaluation was comprehensive and multi‐faceted (Figure 2). The pilot was primarily evaluated using five individual semi‐structured interviews. These were conducted using an interview schedule of four open questions, with further probing questions as led by each conversation. The transcription function of Microsoft Teams was utilised. Both researchers independently coded all transcripts, before comparing codes and developing themes together, leading to a comprehensive thematic analysis of the session. The flexibility of the thematic analysis approach was valued and chosen to ‘provide a rich and detailed, yet complex, account of [the] data’ [9, p. 78].

Additionally, each researcher completed a written self‐reflection. The pilot workshop was recorded, after gaining informed consent, and was reviewed by the research supervisor, who provided written comment. After the session, each participant was asked ‘what have you learnt?’. Responses were reviewed against the session learning objectives. By combining these methods of data collection, themes and areas for improvement could be inductively identified [9].

## 4 Results

**FIGURE 3:**
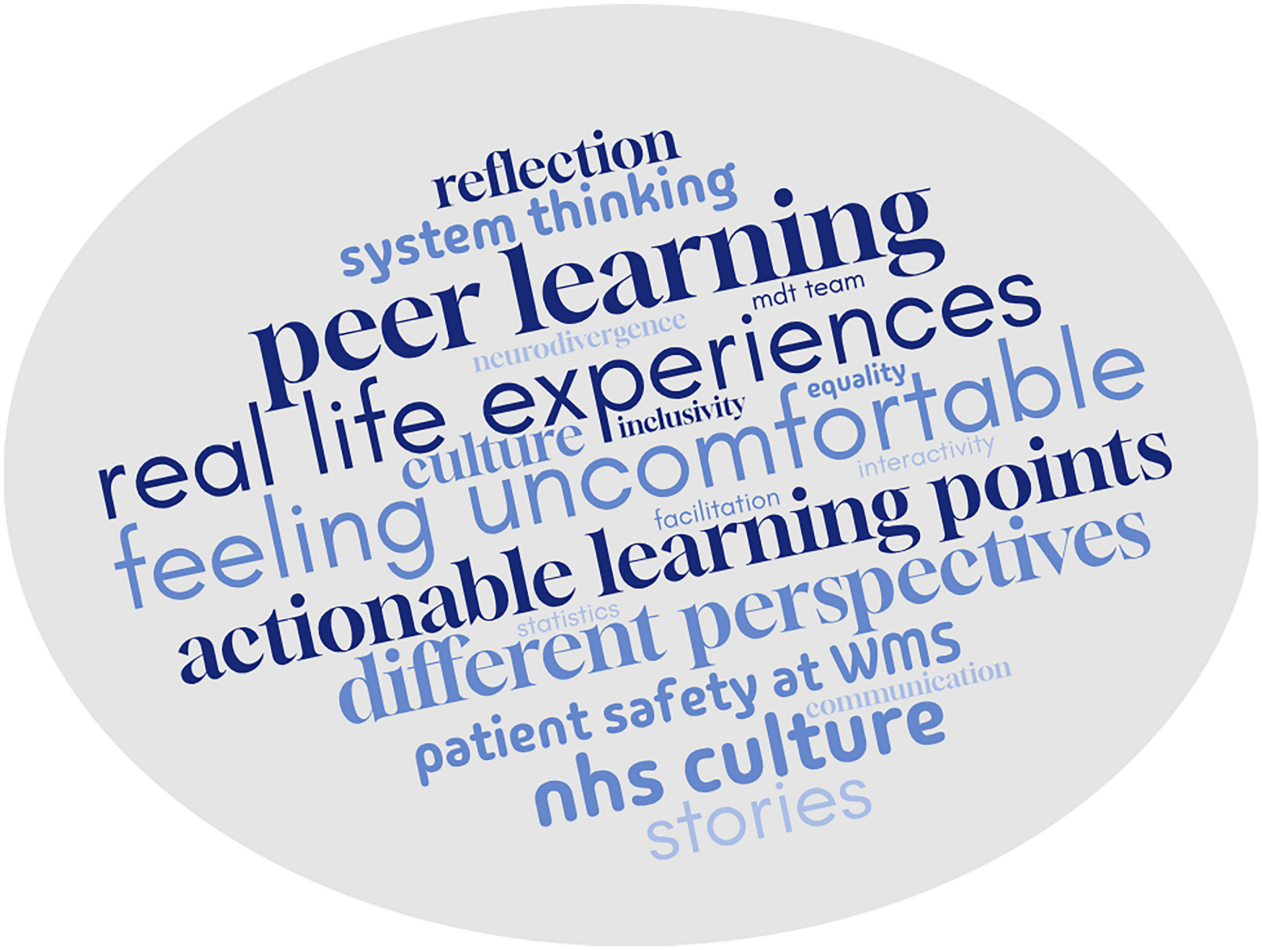
Word cloud of themes identified during the evaluation.

Thematic analysis of interview data from the pilot workshop identified several themes, shown in Figure 3.

### 4.1 Importance of Patient Voice

Incorporating real patient experience was highly valued, with all students commenting how they appreciated this aspect of the core activities. Students indicated how the use of patient stories made the content impactful and provided a tangible basis for discussion.

“[S]tatistics are great, but […] it's always better when you hear it from personal story.”

“I think doing something like [the game‐show inspired activity], trying to think through the eyes of a patient. I think it's a really valuable exercise.”

### 4.2 Feeling Uncomfortable

Several participants expressed feelings of discomfort after the session. These feelings related to the content and the format of the ‘In Your Shoes’ activity. Discussing patient safety can be difficult and evoke memories of previous events. Presenting unfamiliar content to a group can also be fear‐inducing.

Whilst the session elicited negative emotions, this is not necessarily counterintuitive to learning. Kumagai et al state that ‘discomfort and even conflict are essential to learning because they prompt self‐reflection on one's own identities, values, experiences and worldviews’ [10, p. 320]. They further assert ‘[e]xposure to discomfort not only is unavoidable in the practice of medicine but may be crucial to personal and professional moral development’ [10, p. 318]. Overall, participants reflected that despite discomfort, the activities and early introduction to patient safety useful and thought‐provoking.

“It made me feel concerned […] as though there are a lot of things that can obviously go wrong.”

“I felt a bit embarrassed doing the acting.”

Responding to this feedback, a practical advice and signposting section on reporting a safety incident was added. Faculty members were also available during the workshops, should an individual need support.

### 4.3 Peer Learning

The value of peer learning was cited by multiple participants. Our students were graduate entry and consequently had a range of professional and educational backgrounds, including nurses and healthcare assistants. At points, discussion was open to allow people to share their experiences of patient safety, both professionally and personally. Shah et al comment that peer‐learning can increase the perceived relevancy of the subject matter and provide a safe space for personal anecdotes, leading to a likely increased awareness of patient safety trends that affect the provision of care [11].

“I think hearing about personal experiences can really, really help and [you] can ask questions like there and then.”

Additionally, in feedback following the workshop's full cohort delivery, some students commented positively on the peer‐led nature of the session. Near‐peer teaching appeared to create an environment in which students felt more at ease sharing their experiences. We believe this is particularly important for topics such as patient safety, where there may be fear of blame or judgement.

“It being facilitated by another student made it a much more relaxed and interactive environment that I found I was more able to engage with.”

Overall, 88% of 117 student respondents rated the workshop at least a score of 4 out of 5, and it has since been integrated into the curriculum.

## 5 Implications

### 5.1 Greater Scope for Medical Student Involvement in Curriculum Design

This project was a valuable exercise in developing a session on a core but feared topic of clinical learning, directed by both patient and student priorities. Simultaneously, this project demonstrated how there is significant scope for medical students to contribute meaningfully to the curriculum, particularly when supported by robust multi‐modal evaluation techniques to develop and refine learning materials.

The encouraging ratings from the full cohort sessions and the decision by Warwick Medical School to integrate the workshop into the curriculum, give weight to the idea that the skills of students, in developing peer learning materials, may be underutilised across medical schools. Having a unique perspective to understand the clinical experiences, concerns, and vulnerabilities of students, with support, medical students are capable of developing materials particularly tailored to addressing the needs of their peers.

### 5.2 The Potential for Medical Students as Drivers of Improved Patient Safety

Moreover, medical and other healthcare students have a unique position, often being the only additional person present with a healthcare professional and a patient. If the student feels empowered to speak out, should they encounter something amiss, this viewpoint enables the student to be a powerful driver of patient safety.

Going forwards, we hope the workshop serves to dispel fear of a ‘blame culture’ and provides practical knowledge on the channels available to report patient safety concerns. We recognise the limitations of our approach, particularly in needing a set of engaged, enthusiastic and well‐trained student facilitators. Nevertheless, we hope through this investment, the workshop serves as a safe place for students to gain greater confidence to seek support when they themselves encounter a patient safety issue. For future research, it may be insightful to identify whether there is an increase in student reporting, following this patient safety training.

## Author Contributions

**Kirsty Matthews:** conceptualization, funding acquisition, writing – original draft, visualization, methodology, project administration, resources, data curation, formal analysis, investigation, writing – review and editing, validation. **Millie Pierce:** conceptualization, funding acquisition, writing – original draft, writing – review and editing, visualization, project administration, resources, data curation, formal analysis, investigation, methodology, validation.

## Ethics Statement

Course‐delegated University of Warwick   Research Ethics Committee approval was gained for this study.

## Conflicts of Interest

The authors declare no conflicts of interest.

## Data Availability

The data that support the findings of this study are available from the corresponding author upon reasonable request.
